# Regulation and function of the gill corticotropin-releasing factor system during osmoregulatory disturbances in Atlantic salmon

**DOI:** 10.1242/jeb.248168

**Published:** 2025-01-28

**Authors:** Brett M. Culbert, Emma Mossington, Stephen D. McCormick, Nicholas J. Bernier

**Affiliations:** ^1^ Department of Integrative Biology, University of Guelph, 50 Stone Road East, Guelph, ON, Canada, N1G 2W1; ^2^ US Geological Survey, Eastern Ecological Science Center, S.O. Conte Anadromous Fish Research Laboratory, Turners Falls, MA 01376, USA; ^3^ Department of Biology, University of Massachusetts, Amherst, Amherst, MA 01003, USA

**Keywords:** Angiogenesis, Endothelial permeability, Immune regulation, RNA-Seq, Smoltification, Seawater transfer

## Abstract

While corticosteroids, including cortisol, have conserved osmoregulatory functions, the relative involvement of other stress-related hormones in osmoregulatory processes remains unclear. To address this gap, we initially characterized the gill corticotropin-releasing factor (CRF) system of Atlantic salmon (*Salmo salar*) and then determined: (1) how it is influenced by osmotic disturbances; (2) whether it is affected by cortisol; and (3) which physiological processes it regulates in the gills. Most CRF system components were expressed in the gills, with CRF receptor 2 (*crfr2a*), CRF binding protein (*crfbp1* and *crfbp2*) and urocortin 2 (*ucn2a*) being the most abundant. The development of seawater tolerance in migratory juveniles (i.e. smolts) was associated with a general transcriptional upregulation of CRF ligands, but transcript levels of *crfr2a*, *crfbp2*, *crfb2* and *ucn2a* decreased by ∼50% following seawater transfer. Accordingly, transfer of seawater-acclimated fish into freshwater increased *crfr2a* and *ucn2a* levels. Cortisol treatment of cultured gill filaments had marked effects on the CRF system; however, these effects failed to fully replicate changes observed during *in vivo* experiments, suggesting direct contributions of the gill CRF system during osmotic disturbances. Indeed, activation of the CRF system in cultured filaments from freshwater-acclimated (but not seawater-acclimated) salmon had transcriptional effects on several physiological systems (e.g. endothelial permeability, angiogenesis and immune regulation) which involved contributions by both CRF receptor subtypes. Overall, our results indicate that the gill CRF system is more active in hypoosmotic environments and directly contributes to the coordination of physiological responses following osmotic disturbances.

## Teaser

**Summary:** A combination of *in vivo* and *in vitro* studies reveals that the corticotropin-releasing factor system serves osmoregulatory functions in the gills of Atlantic salmon.

## INTRODUCTION

An animal's ability to appropriately regulate levels of ions and water in its body (i.e. osmoregulate) is imperative for its survival. This is especially challenging for anadromous fishes which migrate between freshwater (FW) and seawater (SW) as part of their natural life history (Shrimpton, 2013; Zydlewski and Wilkie, 2013) owing to the distinct osmoregulatory challenges associated with living in FW versus SW. In FW, fish are hyperosmotic to their environment and must actively absorb ions and excrete water to survive, whereas fish in SW must actively excrete ions and conserve water as they are hypoosmotic to their environment (Takei et al., 2014). To survive in these contrasting environments, fish modify the abundance and/or activity of various channels, pumps and transporters located on osmoregulatory tissues, such as the kidney, intestine and gills (Kültz, 2015; Larsen et al., 2014; Zimmer and Perry, 2022). These modifications either occur directly in response to changes in the surrounding osmotic environment [i.e. osmosensing (Fiol and Kültz, 2007; Kültz, 2012)] or are triggered by environmental changes (e.g. daylength) which precede seasonal transitions between osmotic environments [e.g. migrations between FW and SW (Shrimpton, 2013; Zydlewski and Wilkie, 2013)]. For example, the seasonal acquisition of SW tolerance in juvenile salmonid fishes during the transition from non-migratory parr into migratory smolts (i.e. smoltification) is associated with numerous changes in osmoregulatory systems while fish remain in FW (Loretz et al., 1982; McCormick et al., 2013; Sundh et al., 2014). Many of these changes are hormonally mediated (McCormick, 2013), and while several hormones are involved in coordinating osmoregulatory processes in fishes [e.g. growth hormone, prolactin and insulin-like growth factor 1 (Mancera and McCormick, 2007; Takei et al., 2014)], the osmoregulatory actions of the stress-related corticosteroid hormone cortisol are critical to the ability of fish to move between FW and SW.

Cortisol is generally associated with stress responses (Best et al., 2024), but it also serves to control osmoregulatory functions in euryhaline fishes. In FW, cortisol interacts with prolactin to promote hyper-osmoregulatory mechanisms including increased ion uptake by the gills (Mancera and McCormick, 2007; Takei et al., 2014). In contrast, cortisol promotes hypo-osmoregulatory mechanisms during SW acclimation, such as stimulating salt secretion mechanisms in the gills (Kiilerich et al., 2007; Pelis and McCormick, 2001) and water absorption by the intestine (Cornell et al., 1994; Veillette et al., 1995). Indeed, treatment with cortisol receptor antagonists impairs osmoregulatory processes during acclimation to both FW (Scott et al., 2005) and SW (Marshall et al., 2005; Shaw et al., 2007; Veillette et al., 2007). Consistent with its osmoregulatory functions, circulating cortisol levels increase ∼10-fold (Culbert et al., 2022; McCormick, 2013) and the abundance of glucocorticoid receptors changes in osmoregulatory tissues [e.g. the gills (Mizuno et al., 2001; Nilsen et al., 2008; Shrimpton and McCormick, 1998; Shrimpton et al., 1994)] during the parr-to-smolt transformation in salmonids. However, despite the important osmoregulatory role of cortisol, the potential osmoregulatory involvement of other stress-related hormone systems has received far less attention.

The corticotropin-releasing factor (CRF) system – consisting of five peptide ligands [CRFa, CRFb, urotensin 1 (UTS1) and urocortin 2 and 3 (UCN2 and UCN3)], two receptors (CRFR1 and CRFR2) and a binding protein (CRFBP) in teleosts – is primarily known for its central role in controlling cortisol synthesis via the regulation of the hypothalamic–pituitary–adrenal/interrenal axis (Best et al., 2024). However, the CRF system also has direct physiological roles in other tissues. For instance, activation of the CRF system in mammals increases the permeability of both the endothelium and epithelium by influencing the expression of tight junction components such as cadherins, claudins and occludins (Wan et al., 2013; Yu et al., 2013; Yue et al., 2017). The barrier functions of the CRF system have been most thoroughly studied in the gastrointestinal tract of rodents (Stengel and Taché, 2009), where activation of CRF receptor 1 (CRFR1) results in dose-dependent increases in permeability and corresponding elevations in fecal fluid content (Larauche et al., 2009; Liu et al., 2016; Santos et al., 2008; Saunders et al., 2002). The involvement of the CRF system in osmoregulatory processes appears to have occurred early in the evolution of metazoans because the invertebrate analogue of the CRF system – the diuretic hormone 44 (DH44) system (Cardoso et al., 2014; Elphick et al., 2018; Hwang et al., 2013) – also affects these processes. Specifically, DH44 elevates cAMP levels in principal cells of the Malpighian tubules, which promotes the secretion of Na^+^, K^+^ and water (Cabrero et al., 2002; Cannell et al., 2016; Kataoka et al., 1989). These actions are thought to be driven by DH44-mediated changes in V-type H^+^-ATPase and Na^+^/K^+^/Cl^−^-cotransporter (NKCC) activity (Coast, 2007; Larsen et al., 2014), as well as changes in paracellular permeability (Clark et al., 1998). While considerably less research has been conducted on the osmoregulatory actions of the CRF system in fishes, a series of pioneering studies reported that the CRF system – specifically, UTS1 – can influence the movement of water and/or ions across the bladder (Loretz et al., 1981), opercular membrane (Loretz et al., 1981), intestine (Mainoya and Bern, 1982), kidney (Chan, 1975) and skin (Marshall and Bern, 1979, 1981). Additionally, more recent studies have reported that transcript abundance of different components of the CRF system in the gills of Mozambique tilapia [*Oreochromis mossambibus* (Aruna et al., 2012)] and black porgy [*Acanthopagrus schlegelii* (Aruna et al., 2021)] are responsive to salinity changes. However, the mechanistic basis for the osmoregulatory actions associated with the CRF system has yet to be studied in any species of fish.

In the current study, we evaluated how the gill CRF system of Atlantic salmon (*Salmo salar*) is regulated by the osmotic environment and what physiological role(s) this system serves in the gills. We focused on the gills because they are a key osmoregulatory tissue in fish, and previous work has implicated a role for the CRF system during osmotic disturbances in the gills (Aruna et al., 2012, 2021). Similarly, we used Atlantic salmon because they are anadromous (Zydlewski and Wilkie, 2013) and the ability to tolerate changes in the osmotic environment is therefore ecologically relevant and directly linked to their natural life history. We initially characterized which components of the CRF system were expressed in the gills and then determined how expression of the CRF system is affected by ontogenetic shifts in SW tolerance [i.e. the transition from a pre-migratory parr into a migratory smolt (McCormick, 2013)] and changes in the surrounding osmotic environment (FW-to-SW or SW-to-FW transfer). We then performed a series of *in vitro* experiments using cultured gill filaments to determine: (1) whether changes in CRF system abundance are due to interactions with cortisol; (2) which physiological systems in the gills are regulated by the CRF system in FW- versus SW-acclimated fish; and (3) which CRF receptor(s) mediates these effects.

## MATERIALS AND METHODS

### Experimental animals and housing

Experiment 1 took place at the US Geological Survey (USGS) S.O. Conte Anadromous Fish Research Laboratory (Turners Falls, MA, USA) using juvenile Atlantic salmon (*Salmo salar* Linnaeus 1758) that were obtained from the Kensington State Hatchery (Kensington, CT, USA) in the autumn of 2018. Fish were held in 1.7 m diameter tanks that were supplied with flow-through ambient Connecticut River water at a flow rate of 4 l min^−1^ and tanks were continuously aerated. Fish were maintained under the natural photoperiod and were fed to satiation (BioOregon, Westbrook, ME, USA) using automatic feeders. In December of 2018, fish were separated by size into parr and pre-smolt groups as described previously (Culbert et al., 2022). Each group of fish was maintained in duplicate tanks containing ∼100 fish and all fish experienced identical temperature regimes throughout the experiment. All fish rearing and sampling protocols were carried out in accordance with USGS institutional guidelines and protocol LSC-9096 that was approved by the USGS Eastern Ecological Science Center Institutional Animal Care and Use Committee.

For all other experiments, juvenile Atlantic salmon were acquired from the Normandale Fish Culture Station (Vittoria, ON, Canada) and experiments occurred in the Hagen Aqualab at the University of Guelph (Guelph, ON, Canada). Fish used for experiments 2, 3, and 4 originated from an anadromous source population (LaHave River, NS, Canada) which has been bred in captivity since 1989. Fish used for experiment 5 originated from a landlocked source population (Sebago Lake, ME, USA) which has been bred in captivity since 1999. While differences in osmoregulatory physiology have been observed between landlocked and anadromous Atlantic salmon populations (e.g. McCormick et al., 2019), smolt development in landlocked populations is only diminished (not absent), suggesting that these differences are likely to be limited. All fish were maintained in 6 foot (∼1.8 m) diameter fibreglass tanks (∼2000 l) that were supplied with aerated, flow-through well water maintained at 12°C and were kept on a 12 h light:12 h dark photoperiod regime. Fish were fed to satiation 3 times per week with commercial pellets (Blue Water Fish Food, Guelph, ON, Canada). A stocking density of ∼100 fish per tank was maintained and fish were kept under these conditions for several months prior to starting experiments. All procedures were carried out in accordance with the Canadian Council for Animal Care guidelines for the use of animals in research and teaching and were approved by the University of Guelph's Animal Care Committee (AUP #4123).

### Experimental design

#### Experiment 1: regulation of the gill CRF system during smoltification and FW-to-SW transfer

Parr [*N*=84; fork length (FL): 11.1±0.1 cm, mass: 14.0±0.38 g; mean±s.e.m.] and smolts (*N*=84; FL: 16.5±0.1 cm, mass: 44.8±1.0 g) were sampled on 19 February, 1 April, 6 May and 15 Ju1y 2019 (*N*=12 per group per time point). Fish were kept at ambient temperature (2–4°C) through the winter and water temperature was increased by 1°C per day to 8–9°C beginning on 15 February. This temperature was maintained throughout the spring so that all sampling points would be at identical temperatures. After 30 May, fish experienced normal summer temperatures (maximum of 18.4°C) until water temperature was decreased again (by 1°C per day to 9–10°C) on 3 July. This temperature regime was chosen to allow for seasonal temperature increases in late winter and summer, but still allow sampling to occur at the same temperature. Based on previous studies of thermal acclimation in fishes (Schulte et al., 2011), we believe that these temperature changes would have a minimal impact on the parameters measured in this study. To avoid potential tank effects, fish from each group (parr or smolts) were sampled from two separate tanks at each time point (*N*=6 per tank). To determine the response of fish following SW exposure, groups of parr and smolts were placed into six 1 m diameter tanks (three tanks of parr and three tanks of smolts; *N*=12 per tank) containing 28 ppt (the highest salinity that parr can survive) recirculating SW during the week of 6 May 2019. These tanks were held at 8.5–9.5°C and contained particle, biological and charcoal filtration, as well as continuous aeration. Fish were fed to satiation every day, but food was withheld the day prior to samplings. Tanks of parr and smolts were sampled after 24, 96 or 240 h of SW exposure. All fish were killed via terminal anaesthesia using NaHCO_3_ (12 mmol l^−1^)-buffered MS-222 (100 mg l^−1^; pH 7.0) after which FL and mass were recorded. Blood was collected from the caudal vasculature using a 1 ml ammonium heparinized syringe, spun at 3200 ***g*** for 5 min at 4°C, and plasma was collected for later measurement of cortisol and osmolality. The second gill arch was removed, the gill filaments were trimmed from ceratobranchials, and filaments were collected for later RNA extraction and quantitative polymerase chain reaction (qPCR). All samples were immediately flash frozen in dry ice prior to being stored at −80°C.

As an initial determination of which components of the CRF system are expressed in the gills, we conducted qPCR on three separate pools of cDNA that each contained equal amounts of cDNA originating from a parr and a smolt that were sampled in February. Values for each transcript were corrected for primer efficiency and were expressed relative to the abundance of *ucn3*, which was the CRF system component with the lowest levels that consistently amplified. Note that Atlantic salmon have two paralogues of each component of the CRF system owing to the salmonid-specific genome duplication (Lien et al., 2016). Therefore, we measured individual paralogues of all CRF system components, except for UCN3, which shares 98% identity across paralogues.

#### Experiment 2: regulation of the gill CRF system during SW-to-FW transfer

Post-smolt Atlantic salmon (*N*=28; FL: 37.8±0.5 cm, mass: 537.8±24.0 g) were held in a recirculating tank which contained ∼2000 l of SW (33 ppt; Instant Ocean Sea Salt) for approximately 6 months. This tank was continuously aerated with an air stone and was equipped with both particle and biological filtration, as well as UV sterilization. At the start of the experiment, *N*=10 salmon were immediately sampled while the remaining fish were split among two ∼500 l tanks containing aerated, flow-through well water. After either 24 or 96 h in FW, fish (*N*=9 per group) were terminally anaesthetized using phenoxyethanol (0.2%) and their plasma (for cortisol and osmolality) and gill filaments (for qPCR) were collected as described above. We also sampled FW-acclimated salmon (*N*=10; FL: 39.4±1.0 cm, mass: 581.3±42.3 g) for comparison (see ‘Experimental animals and housing’, above, for housing conditions).

#### Experiment 3: regulation of the CRF system by cortisol in cultured gill filaments

Juvenile Atlantic salmon (*N*=8; FL: 13.3±0.6 cm, mass: 37.8±1.3 g) held in FW were rapidly killed via cerebral concussion followed by spinal severance. Afterwards, fish were weighed and measured, and the second gill arch on each side of the fish was collected. Both arches were placed into minimum essential medium with Hanks' Balanced Salts (MEM; Gibco, product 11575032) that was supplemented with 500 U ml^−1^ penicillin/streptomycin (Gibco, product 15140122), 4 mg ml^−1^ bovine serum albumin (BSA) and 25 mmol l^−1^ Hepes [final pH of 7.55; as previously described (McCormick and Bern, 1989)]. The distal portion of all filaments (just after the end of the septum) was removed from both arches and filaments were mixed and randomly placed into wells of a 24-well culture plate that contained 0.5 ml of MEM. Filaments were then incubated with shaking for 2 h in MEM solution at 12°C, after which the MEM solution was replaced with one of the treatment solutions. Treatment solutions consisted of vehicle alone (MEM with 0.1% ethanol), cortisol alone (MEM with 10 μg ml^−1^ of cortisol), or a combination of cortisol and the glucocorticoid receptor antagonist RU486 (MEM with 10 μg ml^−1^ of cortisol and 10 μg ml^−1^ of RU486). Twenty-four hours later, filaments were collected and frozen on dry ice to facilitate later RNA extraction and qPCR analysis. Specifically, we measured levels of several components of the gill CRF system, including peptides (*crfa1*, *crfa2*, *ucn2a1* and *ucn3*), a binding protein (*crfbp2*) and receptors (*crfr1a*, *crfr1b*, *crfr2a* and *crfr2b*). Additionally, as confirmation that our cortisol and receptor antagonist doses were appropriate, we initially assessed treatment effects on transcript abundance of the SW isoform of Na^+^/K^+^-ATPase subunit alpha-1 (*nkaα1b*), which is transcriptionally upregulated by cortisol via glucocorticoid receptor activation (Kiilerich et al., 2007). Preliminary experiments showed that RU486 alone (10 µg ml^−1^) did not affect transcript levels of any targeted gene. Cortisol (product H4001) and RU486 (product M8046) were both purchased from Sigma-Aldrich (Oakville, ON, Canada).

#### Experiment 4: determination of the transcriptional roles of the CRF system in cultured gill filaments using RNA-Seq

Salmon that were acclimated to either FW (*N*=8; FL: 33.9±0.9 cm; mass: 385.5±45.0 g) or SW (*N*=8; FL: 33.9±0.9 cm; mass: 393.1±24.6 g) were processed as for experiment 3 above, with the exception that plasma was also collected from these fish to allow measurement of plasma cortisol and osmolality. Filaments were incubated in MEM that contained 0.1% DMSO (vehicle control), 1 nmol l^−1^ of CRFa2 (CRFa) or 1 nmol l^−1^ of UCN3a/b (UCN3) for 24 h. After this period, filaments were collected, flash frozen on dry ice and stored at −80°C until RNA was extracted to allow for RNA-Seq analysis (see ‘RNA-Seq’, below, for further details). Whereas CRFa2 activates both CRFR1 and CRFR2 (Hosono et al., 2015), UCN3 is highly selective for CRFR2 (Manuel et al., 2014). The CRFa2 and UCN3a/b peptides were custom synthesized by GenScript Biotech (Piscataway, NJ, USA) according to the deduced amino acid sequences for Atlantic salmon in GenBank (see Table S1). Note that the peptide sequences of the two paralogues of UCN3 are 100% identical in Atlantic salmon. Hormone doses were selected based on preliminary experiments showing that 1 nmol l^−1^ CRFa2 stimulated PKA activity [a primary effector of CRFR activation (Lovejoy et al., 2014)] to the greatest extent in cultured gill filaments based on levels of PKA-substrate phosphorylation as determined by SDS-PAGE and western blotting (see Supplementary Materials and Methods and Fig. S1 for additional details).

We also conducted qPCR (see ‘RNA isolation and qPCR’, below) using cDNA that was synthesized from the same RNA that was sent for RNA-Seq to confirm that we could replicate the RNA-Seq results via qPCR with gene-specific primers (Table S1). Specifically, we focused on several of the most abundant and differentially expressed genes (*cah15b*, *cldn5b*, *dtx4a*, *igfbp5a*, *hsp90aa1*, *hsp90aa2*, *mylk*, *serpine1*, *timp3*, *trpv6*, *vcam1*, *vgf*; see Table 1 for full gene names), allowing us to further explore the transcriptional effects mediated by the gill CRF system in the follow-up experiment described below. Lastly, we evaluated transcript abundance of all CRFR paralogues (*crfr1a*, *crfr1b*, *crfr2a* and *crfr2b*) in FW versus SW vehicle groups to evaluate the effects of culture in hypoosmotic culture media (∼298 mOsm).

**Table 1.** Transcripts in the gill filaments of freshwater-acclimated Atlantic salmon (*Salmo salar*; *N*=8) that were significantly affected by treatment with either corticotropin-releasing factor a2 (CRFa) or urocortin 3 (UCN3) based on RNA-Seq analysis

Ensembl ID	Symbol	Gene name	log_2_ FC	log_2_ cpm	*P*-value
CRFa	* *				
ENSSSAG00000097755	*vcam1*	vascular cell adhesion molecule 1	0.80	3.72	<0.001
ENSSSAG00000081488	*csrnp3*	cysteine and serine rich nuclear protein 3	0.43	1.99	0.04
ENSSSAG00000042311	*il23r*	interleukin-23 receptor alpha	0.41	2.94	0.03
ENSSSAG00000071823	*ccl19*	chemokine (C-C motif) ligand 19a, tandem duplicate 1	0.41	4.15	0.046
ENSSSAG00000088363	*igfbp5a*	insulin-like growth factor-binding protein 5a	0.40	3.53	0.01
ENSSSAG00000059452	*il17r*	interleukin-17 receptor E-like	0.38	2.62	0.04
ENSSSAG00000069972	*id1*	DNA-binding protein inhibitor ID-1-like	0.37	5.58	0.02
ENSSSAG00000007617	*trpv6*	transient receptor potential cation channel, subfamily V, member 6	0.36	3.78	0.02
**ENSSSAG00000071373**	***mylk***	**myosin light chain kinase**	**0.36**	**5.60**	**<0.001**
ENSSSAG00000095386	*ctnnd2a*	catenin (cadherin-associated protein), delta 2a	0.34	2.85	0.04
ENSSSAG00000002470	*ank3a*	ankyrin 3a	0.34	4.97	0.002
ENSSSAG00000000188	*si1l1*	signal-induced proliferation-associated 1-like protein 1	0.30	4.72	0.046
ENSSSAG00000108925	*col4a4*	collagen, type IV, alpha 4	0.28	6.98	0.02
ENSSSAG00000063834	*hsp90aa2*	heat shock protein 90, alpha, class A member 1	−0.28	8.36	0.02
ENSSSAG00000067399	*sema3ga*	semaphorin 3Ga	−0.30	5.60	0.03
ENSSSAG00000043212	*tm4sf18*	transmembrane 4 L six family member 18	−0.31	4.07	0.046
ENSSSAG00000068003	*vgf*	VGF nerve growth factor inducible	−0.32	5.17	0.01
ENSSSAG00000064153	*ets1*	protein C-ets-1	−0.34	4.92	0.006
ENSSSAG00000069027	*tfpi2*	tissue factor pathway inhibitor 2	−0.36	4.59	0.04
ENSSSAG00000042708	*hsp90aa1*	heat shock protein 90, alpha, class A member 1	−0.36	5.58	0.01
ENSSSAG00000047458	*cldn5b*	claudin 5b	−0.38	7.27	0.001
ENSSSAG00000118402	*timp3*	metalloproteinase inhibitor 3	−0.39	7.75	0.002
ENSSSAG00000097633	*lratd2*	LRAT domain containing 2	−0.39	3.51	0.02
ENSSSAG00000105982	NA	uncharacterized	−0.42	2.19	0.02
ENSSSAG00000108981	*lncRNA*	Long non-coding RNA	−0.42	2.95	0.02
ENSSSAG00000104087	*bag3*	BCL2 associated athanogene 3	−0.43	2.88	0.02
ENSSSAG00000073073	*inavaa*	innate immunity activator a	−0.43	3.07	0.046
ENSSSAG00000055981	*vegfc*	vascular endothelial growth factor c	−0.50	1.88	0.04
ENSSSAG00000091334	*tspan3*	tetraspanin 3	−0.52	2.56	0.003
ENSSSAG00000102772	*fosb*	FBJ murine osteosarcoma viral oncogene homolog B	−0.63	2.77	0.03
UCN3					
ENSSSAG00000057907	*cah15b*	carbonic anhydrase 15b	0.45	4.12	0.006
ENSSSAG00000040387	*dtx4a*	deltex 4, E3 ubiquitin ligase a	0.37	3.74	0.04
ENSSSAG00000055178	*abi1*	ABI family, member 3a	0.34	3.58	0.03
**ENSSSAG00000071373**	***mylk***	**myosin light chain kinase**	**0.32**	**5.57**	**0.03**
ENSSSAG00000080970	*crld2*	cysteine-rich secretory protein LCCL domain-containing 2	−0.28	5.30	0.04
ENSSSAG00000068013	*serpine1*	serpin peptidase inhibitor, clade E, member 1	−0.29	5.69	0.04
ENSSSAG00000083881	*foxq1a*	forkhead box Q1a	−0.30	5.25	0.004

Fold-change (FC) and counts per million (cpm) values are both reported on a log_2_ scale and *P*-values are false-discovery rate corrected. Transcripts in bold were significantly different from vehicle-treated filaments for both treatments. NA, not available.

#### Experiment 5: determination of CRF receptor-specific effects in cultured gill filaments

Gill filaments were collected from FW-acclimated salmon (*N*=12; FL: 15.4±0.3 cm, mass: 41.1±2.8 g) as described above. Preliminary dose–response experiments indicated that a 10-fold higher dose of CRFa2 (10 nmol l^−1^) was necessary to elicit similar transcriptional changes in this landlocked source population versus the anadromous source population used for experiment 4 (Fig. S2). As CRFa2 activates both CRFR1 and CRFR2 (Hosono et al., 2015), we treated a subset of filaments with both CRFa2 and antalarmin [a specific antagonist of CRFR1 (Alderman et al., 2018; Webster et al., 1996)] to determine which of the observed transcriptional effects of CRFa2 were specifically mediated by CRFR1. Thus, a subset of filaments from each fish were treated with vehicle alone (MEM with 0.1% DMSO), CRFa2 alone (MEM with 10 nmol l^−1^ of CRFa2), or a combination of CRFa2 and antalarmin (MEM with 10 nmol l^−1^ of CRFa2 and 100 nmol l^−1^ antalarmin). All other details were identical to the protocol described for experiment 3 above and transcript abundance was assessed using qPCR (as described in ‘RNA isolation and qPCR’, below). We only evaluated genes from experiment 4 in which significant differences were detected using both RNA-Seq and qPCR (*igfbp5a*, *hsp90aa1*, *mylk*, *serpine1*, *timp3*, *trpv6*, *vcam1*; see Table 1 for full gene names). Antalarmin hydrochloride was purchased from Cayman Chemical (product 15147; Ann Arbor, MI, USA).

### Plasma cortisol and osmolality

Plasma osmolality values were determined in duplicate using a vapour pressure osmometer (Vapro 5520, Wescor) and had an intra-assay coefficient of variation (CV) of 8.7%. Circulating cortisol levels were determined using a previously validated direct competitive enzyme immunoassay [EIA (Carey and McCormick, 1998)] in experiment 1, or a commercially available EIA (Neogen, cat. no. 402710) for all other experiments. Intra- and inter-assay variation for cortisol measurement was 9.4% and 12.4% CV, respectively.

### RNA isolation and qPCR

Gill filaments were homogenized in TRIzol reagent (Invitrogen) using a Precellys Evolution tissue homogenizer (Bertin Instruments, Montigny-le-Bretonneux, France). Following the manufacturer's protocol, total RNA was extracted, and its quantity and purity were assessed using a NanoDrop 2000 spectrophotometer (Thermo Fisher Scientific, Mississauga, ON, Canada). We then treated 1 µg of RNA with DNase (DNase 1; Thermo Fisher Scientific) and reverse transcribed cDNA using a high-capacity cDNA reverse transcription kit (Applied Biosystems, Waltham, MA, USA). We then performed qPCR using a CFX96 system (BioRad, Hercules, CA, USA) with SYBR green (SsoAdvanced Universal; BioRad) and all samples were run in duplicate. Negative controls, including no-template controls (where cDNA was replaced with water) and no-reverse transcriptase controls (where reverse transcriptase was replaced with water during cDNA synthesis) were also included. Each reaction contained a total of 20 µl, which consisted of 10 µl of SYBR green, 5 µl of combined forward and reverse primers (0.2 µmol l^−1^ final concentration) and 5 µl of 10× diluted cDNA. Cycling parameters included a 30 s activation step at 95°C, followed by 40 cycles consisting of a 3 s denaturation step at 95°C and a combined 30 s annealing and extension step at 60°C. Melt curve analysis was conducted at the end of each run to confirm the specificity of each reaction. To account for differences in amplification efficiency, standard curves were constructed for each gene using serial dilutions (4×) of pooled cDNA. Input values for each gene were obtained by fitting the average threshold cycle value to the antilog of the gene-specific standard curve, thereby correcting for differences in primer amplification efficiency. To correct for minor variations in template input and transcriptional efficiency, we normalized our data to the geometric mean of the transcript abundance of elongation factor 1α (*ef1α*) and ribosomal protein L13a (*rpl13a*) as reference genes. All data are expressed relative to the mean value of the control group within each experiment (see figure captions for further details).

### RNA-Seq

Extraction of RNA occurred as described in ‘RNA isolation and qPCR’, above, after which it was sent to the Génome Québec Centre of Expertise and Services (Montréal, QC, Canada) for cDNA library preparation and sequencing. The integrity of all RNA was evaluated using a Bioanalyzer 2100 (Agilent Technologies, Santa Clara, CA, USA) and all samples had an RNA integrity number (RIN) greater than 9.7 (9.9±0.1). Using 250 ng of total RNA, mRNA was isolated using NEBNext^®^ Poly(A) Magnetic Isolation Module (New England Biolabs, Ipswich, MA, USA). Stranded cDNA libraries were created using the Illumina Stranded mRNA Prep and sequencing was conducted on all 48 samples using 100 base pair (bp) paired-end reads with a NovaSeq 6000 (Illumina, San Diego, CA, USA). A mean of ∼41,900,000±900,000 paired reads per sample were sequenced (Table S2) and raw sequencing reads were archived with the National Center for Biotechnology Information Sequence Read Archive (accession no.: PRJNA1116796).

Processing of the RNA-Seq data was performed by the Canadian Centre for Computational Genomics (C3G; https://computationalgenomics.ca/) using their in-house RNA-Seq pipeline (https://bitbucket.org/mugqic/genpipes/src/master/genpipes/pipelines/rnaseq/). Briefly, paired reads were joined and checked for quality using FastQC (v0.12.0; Babraham Bioinformatics; https://www.bioinformatics.babraham.ac.uk/projects/fastqc/) and low-quality reads (∼0.02% of all reads) were trimmed using Trimmomatic (v0.39.0; Bolger et al., 2014). Filtered reads from all libraries were aligned to the Atlantic salmon reference genome (Ssal_v3.1, INSDC Assembly; ENSEMBL version 109.31) using STAR (v2.7.8a; Dobin et al., 2013). This resulted in an average alignment rate of 92.1±0.2% across all samples, which provided an average of 13,246±110 transcripts per sample, with a total of 54,224 unique transcripts identified in total. Picard (v3.0.0; Picard Tools) was used to create a single global BAM file for each sample and RNA expression quantification was performed using HTSeq-count (v2.0.2; Anders et al., 2015). Using the edgeR package (v3.42.4; Robinson et al., 2009) in R (v4.3; http://www.R-project.org/), genes were filtered for low expression [a counts per million (cpm) score of ≥3], which resulted in a final count of 22,916 and 24,511 unique transcripts in filaments from FW- and SW-acclimated fish, respectively.

### Statistical analyses

Statistical analyses were performed using R (v4.4.0; http://www.R-project.org/). All data are presented as means±1 s.e.m. and a significance level (α) of 0.05 was used for all tests. Outliers were excluded based on a 2× interquartile range threshold for experiments 1, 2, 3 and 5. When data for these experiments did not meet the assumptions of normality and/or equal variance, data were either log or square-root transformed to meet the assumptions, or analyses were performed using ranked data. Data for experiment 1 were analysed using two-way ANOVA that included group (parr and smolt) and either month (February, April, May and July) or time following SW exposure (FW, 24, 96 and 240 h), as well as the interaction between these factors. All plasma osmolality and plasma cortisol data from experiments 2 and 4, as well as the qPCR data from experiment 2 and the CRFR data from experiment 4, were analysed using either one-way ANOVA or *t*-test, where either time (experiment 2) or treatment group (experiment 4) was included as a fixed factor. As all other qPCR data from experiments 3–5 used filaments from the same fish across all treatment groups, we analysed these data with linear mixed models that included fish ID as a random effect using the ‘lme4’ package (Bates et al., 2015). When significant differences were detected, *post hoc* Tukey's tests were performed using the ‘emmeans’ package (Lenth, 2016). For the RNA-Seq analysis, samples from each treatment group (CRFa or UCN3) were individually compared against vehicle-treated filaments that were collected from the same fish. Specifically, each sample was normalized to its overall library size and differential gene expression testing was performed in edgeR (v3.42.4; Robinson et al., 2009) using generalized linear models (which included treatment and fish ID as factors) followed by *F*-tests. The resulting *P*-values from these analyses were false discovery rate corrected using the Benjamini–Hochberg method within edgeR. Additionally, an effect size of ≥20% fold-change between treatment and vehicle groups was used for all RNA-Seq analyses (i.e. differences of <20% were dropped).

## RESULTS

### Characterization of the gill CRF system

**Fig. 1.**
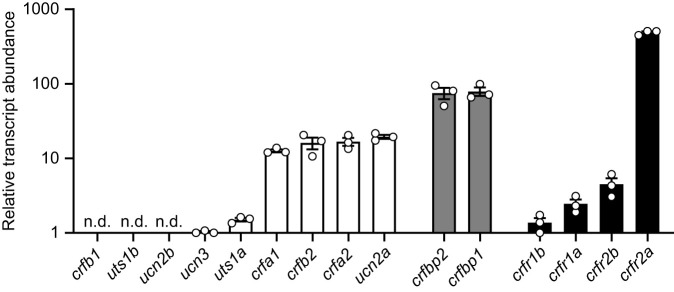
**Characterization of the gill corticotropin-releasing factor (CRF) system.** Relative abundance of individual ligands (white), binding proteins (grey) and receptors (black) of the CRF system in the gills of Atlantic salmon (*Salmo salar*). Bars represent mean abundance (±s.e.m.) of each component relative to *ucn3* (the component with the lowest detectable levels). Each point (*N*=3) represents a pooled sample of gill filaments collected from a parr and a smolt sampled in February (see Materials and Methods, ‘Experimental design’, ‘Experiment 1: regulation of the gill CRF system during smoltification and FW-to-SW transfer’ for further details). Note that data are plotted on a log_10_ scale for visualization purposes. n.d., not detected.

The only components of the CRF system that were not detectable at the transcript level were *crfb1*, *ucn2b* and *uts1b*; all other components consistently amplified (Fig. 1). The most abundant component was *crfr2a* (amplified at ∼25 cycles), which was ∼6 times more abundant than both binding proteins (*crfbp1* and *crfbp2*), 20–30 times more abundant than the most abundant peptides (*crfa1*, *crfa2*, *crfb2* and *ucn2a*) and ∼100–400 times more abundant than all other receptors (*crfr1a*, *crfr1b* and *crfr2b*).

### Seasonal changes in the gill CRF system (parr and smolts)

**Fig. 2.**
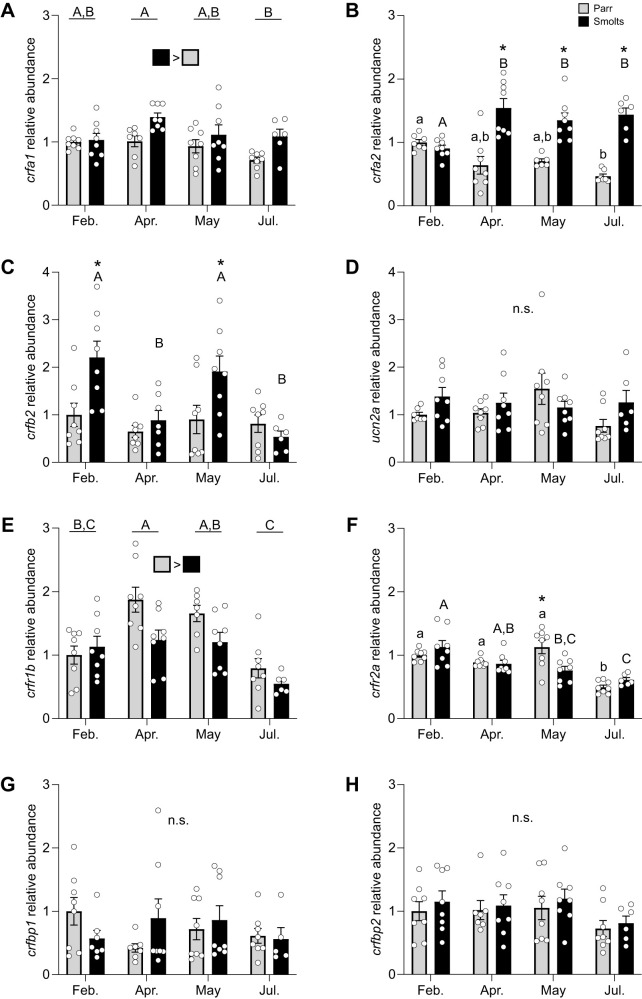
**Changes in gill CRF system components during smoltification.** Seasonal changes in transcript abundance of CRF a1 (*crfa1*; A), a2 (*crfa2*; B) and b2 (*crfb2*; C), urocortin 2a (*ucn2a*; D), CRF receptor 1b (*crfr1b*; E) and 2a (*crfr2a*; F), and CRF binding protein 1 (*crfbp1*; G) and 2 (*crfbp2*; H) in gill filaments of pre-migratory parr (grey) and migratory smolt (black) Atlantic salmon (*S. salar*). Significant differences (*P*<0.05; two-way ANOVA) are depicted using letters (across time: uppercase, within smolts; lowercase, within parr; underlined uppercase, overall time effect), filled squares (between groups across all time points) or asterisks (between groups within a time point). Data are expressed relative to parr in February. Values are means±s.e.m. and individual data points are shown (*N*=5–8). n.s., no significant difference.

Levels of *crfa1* (Fig. 2A; *P*_group_=0.002, *P*_time_=0.03, *P*_group×time_=0.19) and *crfa2* (Fig. 2B; *P*_group_<0.001, *P*_time_=0.45, *P*_group×time_<0.001) were 30–80% higher in smolts compared with parr, especially during all post-February samplings. Similarly, levels of *crfb2* (Fig. 2C; *P*_group_=0.004, *P*_time_<0.001, *P*_group×time_=0.02) were twice as high in smolts versus parr in February and May. No differences were detected for *ucn2a* (Fig. 2D; *P*_group_=0.10, *P*_time_=0.22, *P*_group×time_=0.12). Levels of *crfr1b* (Fig. 2E; *P*_group_=0.008, *P*_time_<0.001, *P*_group×time_=0.08) were ∼25% higher overall in parr compared with smolts but decreased during the summer in both groups. Levels of *crfr2a* (Fig. 2F; *P*_group_=0.62, *P*_time_<0.001, *P*_group×time_=0.001) also decreased seasonally by ∼50% in both groups. However, no changes were detected for either *crfbp1* (Fig. 2G; *P*_group_=0.98, *P*_time_=0.61, *P*_group×time_=0.29) or *crfbp2* (Fig. 2H; *P*_group_=0.27, *P*_time_=0.11, *P*_group×time_=0.96).

As reported in Culbert et al. (2022), plasma osmolality (Table 2) was slightly higher (∼2%) in smolts versus parr through the spring and summer. Plasma cortisol values (Table 2) were also ∼4.7 times higher in smolts than in parr and were 4- to 8-fold higher overall in April than in February, May or July.

**Table 2.** Plasma cortisol and osmolality values for Atlantic salmon (*S. salar*) from experiments 1, 2 and 4

Variable	Exp.		Sampling time				*P*-value	
Plasma cortisol (ng ml^−1^)			Feb.^Y^	Apr.^Z^	May^Y^	Jul.^Y^		
	1	Parr^A^	0.2±0.1	1.8±0.4	0.5±0.1	0.2±0.1	Time	**<0.001**
		Smolts^B^	0.8±0.2	7.2±2.9	2.4±0.6	2.2±0.7	Group	**<0.001**
							Interaction	0.34
			FW	24 h	96 h	240 h		
		Parr	0.5±0.1^a,^*	170.5±25.1^b,^*	18.0±7.8^c^	12.7±4.2^c,^*	Time	**<0.001**
		Smolts	2.4±0.6^y^	35.2±8.6^z^	6.2±1.4^y^	90.7±25.4^z^	Group	0.15
							Interaction	**<0.001**
	2		SW	24 h	96 h	FW		** **
			2.0±0.8^a^	2.4±0.8^a^	0.6±0.5^b^	2.9±1.1^a^	Time	**<0.001**
	4		FW	SW				
			9.4±2.8	4.4±1.1			Group	0.13
Plasma osmolality (mOsm kg^−1^)			Feb.	Apr.	May	Jul.		
	1	Parr^A^	309±5	304±6	302±4	310±4	Time	0.20
		Smolts^B^	315±4	304±5	320±3	312±3	Group	**0.04**
							Interaction	0.14
			FW	24 h	96 h	240 h		
		Parr	302±4^a,^*	456±11^c,^*	342±9^b^	326±6^b,^*	Time	**<0.001**
		Smolts	320±3^yz^	327±3^z^	317±4^yz^	313±2^y^	Group	0.06
							Interaction	**<0.001**
	2		SW	24 h	96 h	FW		
			307±1^b^	301±1^a^	303±1^a^	303±1^a^	Group	**0.001**
	4		FW	SW				
			296±2^a^	313±2^b^			Group	**<0.001**

Values are presented as means±s.e.m. Significant differences are indicated in bold. Lowercase letters indicate differences across time within a group, asterisks indicate a difference between groups within a time point, and uppercase letters indicate an overall difference between groups or across times based on *post hoc* analysis. Note that data from experiment 1 have previously been reported in Culbert et al. (2022).

### Effects of FW–SW transfer on the gill CRF system (parr and smolts)

**Fig. 3.**
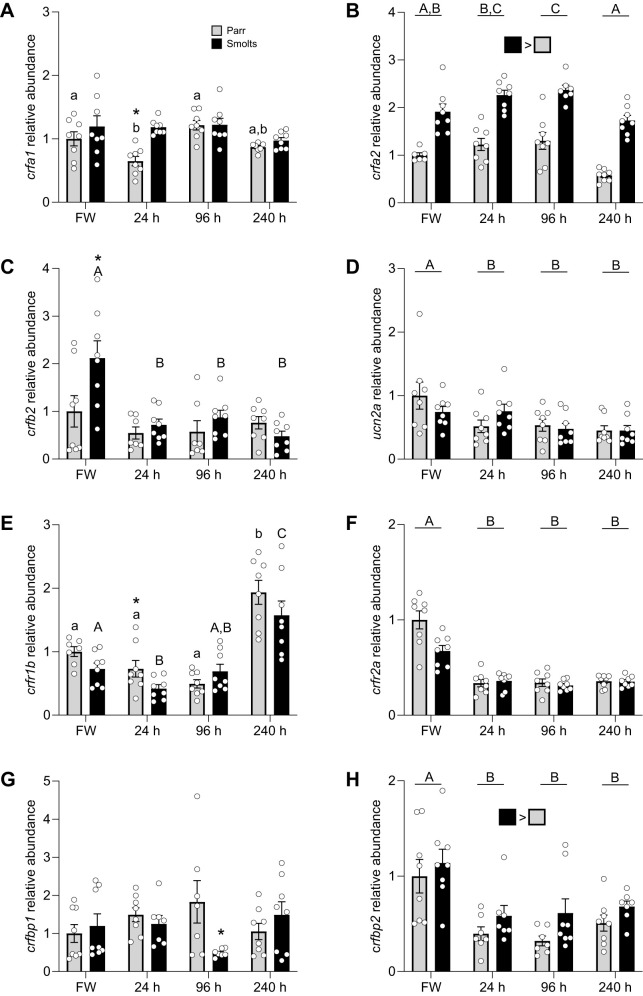
**Effects of freshwater-to-seawater transfer on gill CRF system components.** Changes in transcript abundance of CRF a1 (*crfa1*; A), a2 (*crfa2*; B) and b2 (*crfb2*; C), urocortin 2a (*ucn2a*; D), CRF receptor 1b (*crfr1b*; E) and 2a (*crfr2a*; F), and CRF binding protein 1 (*crfbp1*; G) and 2 (*crfbp2*; H) in gill filaments of pre-migratory parr (grey) and migratory smolt (black) Atlantic salmon (*S. salar*) following seawater (SW; sampled at 24, 96 and 240 h) transfer during peak smoltification in May. Significant differences (*P*<0.05; two-way ANOVA) are depicted using letters (across time: uppercase, within smolts; lowercase, within parr; underlined uppercase, overall time effect), filled squares (between groups across all time points) or asterisks (between groups within a time point). Data are expressed relative to parr in freshwater (FW). Values are means±s.e.m. and individual data points are shown (*N*=6–8).

Levels of *crfa1* (Fig. 3A; *P*_group_=0.002, *P*_time_=0.03, *P*_group×time_=0.19) were ∼25% higher in smolts than in parr and decreased transiently at 24 h after SW transfer in parr (but not smolts). In contrast, levels of *crfa2* (Fig. 3B; *P*_group_<0.001, *P*_time_<0.001, *P*_group×time_=0.81) increased up until 96 h post-transfer after which they decreased by 40% in both groups and were ∼2- to 3-fold higher in smolts compared with parr overall. While levels of *crfb2* (Fig. 3C; *P*_group_=0.03, *P*_time_=0.02, *P*_group×time_=0.02) were ∼2 times higher in smolts prior to SW transfer, *crfb2* abundance decreased by ∼70% in smolts following SW transfer. Similarly, levels of *ucn2a* (Fig. 3D; *P*_group_=0.78, *P*_time_=0.002, *P*_group×time_=0.31) decreased by ∼50% following SW transfer in both parr and smolts. Levels of *crfr1b* (Fig. 3E; *P*_group_=0.06, *P*_time_<0.001, *P*_group×time_=0.03) and *crfr2a* (Fig. 3F; *P*_group_=0.20, *P*_time_<0.001, *P*_group×time_=0.09) showed opposite patterns. While *crfr1b* levels increased 2- to 3-fold after fish had acclimated to SW for 240 h, levels of *crfr2a* decreased by ∼70% immediately following SW transfer. Levels of *crfbp1* tended to be highly variable in both groups (Fig. 3G; *P*_group_=0.26, *P*_time_=0.80, *P*_group×time_=0.02), but levels of *crfbp2* (Fig. 3H; *P*_group_=0.003, *P*_time_<0.001, *P*_group×time_=0.74) decreased by ∼50% in parr and smolts following SW transfer, although levels remained ∼40% higher in smolts across all time points.

As reported in Culbert et al. (2022), plasma osmolality (Table 2) in parr increased by ∼50% 24 h after SW transfer and remained ∼10% higher after 96 or 240 h, while plasma osmolality in smolts was only minimally affected by SW transfer. Plasma cortisol levels (Table 2) increased markedly (340-fold) in parr 24 h after SW transfer, while plasma cortisol levels in smolts were elevated to a lesser degree after 24 h (14-fold) and 240 h (38-fold).

### Effects of SW–FW transfer on the gill CRF system

**Fig. 4.**
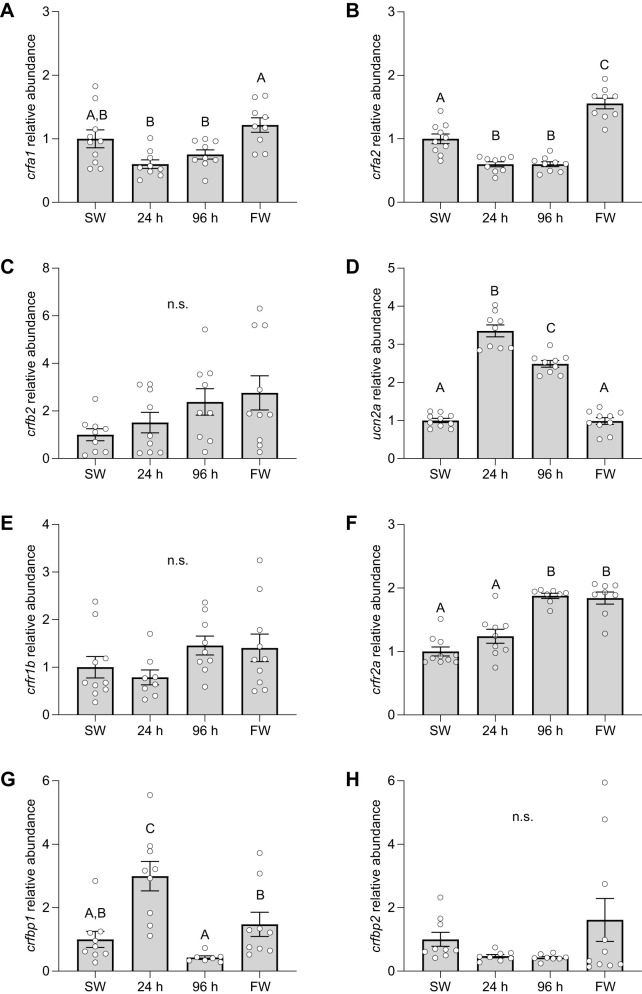
**Effects of SW-to-FW transfer on gill CRF system components.** Changes in transcript abundance of CRF a1 (*crfa1*; A), a2 (*crfa2*; B) and b2 (*crfb2*; C), urocortin 2a (*ucn2a*; D), CRF receptor 1b (*crfr1b*; E) and 2a (*crfr2a*; F), and CRF binding protein 1 (*crfbp1*; G) and 2 (*crfbp2*; H) in gill filaments of Atlantic salmon (*S. salar*) following transfer from SW to FW (sampled at 24 and 96 h). Significant differences (*P*<0.05; one-way ANOVA) are depicted using letters and data are expressed relative to SW-acclimated fish. Values are means±s.e.m. and individual data points are shown (*N*=7–10). The FW group was never transferred to SW. n.s., no significant difference.

Levels of *crfa1* (Fig. 4A; *P*=0.002) and *crfa2* (Fig. 4B; *P*<0.001) both initially decreased by ∼40% following FW transfer, but *crfa2* was 50% higher in FW- versus SW-acclimated fish (with a similar, but non-significant, pattern for *crfa1*). Levels of *crfb2* (Fig. 4C; *P*=0.11) displayed a non-significant increase of ∼150% as fish acclimated to FW. Levels of *ucn2a* (Fig. 4D; *P*<0.001) increased by ∼250% 24 h post-transfer to FW, but these levels gradually decreased such that FW- and SW-acclimated fish were not different. While no differences were detected for *crfr1b* (Fig. 4E; *P*=0.17), *crfr2a* levels doubled as fish acclimated to FW (Fig. 4F; *P*<0.001). Levels of *crfbp1* increased by ∼200% during the initial 24 h after transfer to FW, declined to levels that were ∼50% of SW-acclimated fish after 96 h, and then finally settled such that levels in FW- and SW-acclimated fish were not different from one another (Fig. 4G; *P*<0.001). No significant differences were detected from *crfbp2* (Fig. 4H; *P*=0.15). Plasma osmolality values decreased by ∼1–2% after transfer from SW to FW, while plasma cortisol levels were ∼70% lower 96 h post-transfer (Table 2).

### Effects of cortisol on the CRF system of cultured gill filaments

**Fig. 5.**
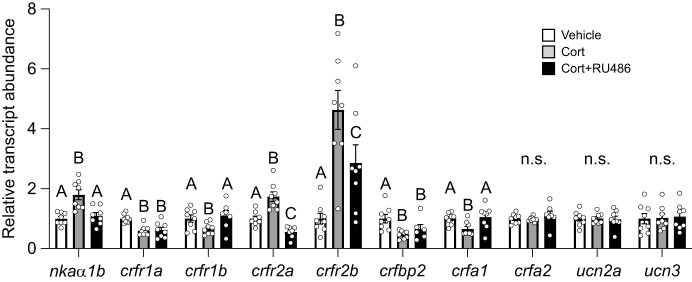
**Effects of cortisol on CRF system components in the gills.** Regulation of Na^+^/K^+^-ATPase α1b (*nkaα1b*), CRF receptor 1a (*crfr1a*), 1b (*crfr1b*), 2a (*crfr2a*) and 2b (*crfr2b*), CRF binding protein 2 (*crfbp2*), CRF a1 (*crfa1*) and a2 (*crfa2*), and urocortin 2a (*ucn2a*) and 3 (*ucn3*) transcript abundance by cortisol in the gills of FW-acclimated Atlantic salmon (*S. salar*). Filaments that were treated with vehicle alone, cortisol (Cort) and cortisol plus the glucocorticoid receptor antagonist RU486 (Cort+RU486) are depicted in white, grey and black, respectively. Significant differences within a gene (*P*<0.05; one-way ANOVA) are depicted using letters and data are expressed relative to vehicle-treated filaments. Values are means±s.e.m. and individual data points are shown (*N*=6–8). n.s., no significant difference.

Cortisol treatment caused an 80% increase in levels of *nkaα1b* (Fig. 5) compared with vehicle-treated filaments (*P*<0.001), but this effect was blocked by RU486 co-treatment because vehicle- and cortisol+RU486-treated filaments did not differ from each other (*P*=0.58). Treatment effects (Fig. 5) were also detected for *crfr1a* (*P*<0.001), *crfr1b* (*P*=0.002), *crfr2a* (*P*<0.001), *crfr2b* (*P*<0.001), *crfbp2* (*P*<0.001) and *crfa1* (*P*=0.002), but not *crfa2* (*P*=0.44), *ucn2a* (*P*=0.93) or *ucn3* (*P*=0.85). Levels of *crfr1a* (*P*=0.002) and *crfr1b* (*P*=0.04) were both reduced by ∼40% following cortisol treatment. However, whereas co-treatment with RU486 blocked the effects of cortisol on *crfr1b* (*P*=0.88 versus vehicle), levels of *crfr1a* in cortisol+RU486-treated filaments remained ∼40% lower than in vehicle-treated filaments (*P*=0.004). Cortisol treatment elevated levels of both *crfr2a* and *crfr2b* (*crfr2a*: ∼75%, *P*<0.001; *crfr2b*: ∼360%, *P*<0.001). However, while levels of *crfr2a* in cortisol+RU486-treated filaments were lower than in either vehicle- (∼45%; *P*=0.03) or cortisol-treated filaments (∼67%; *P*<0.001), RU486 co-treatment only partially blocked the effects of cortisol on *crfr2b*, with cortisol+RU486-treated filaments having levels that were intermediate to those in vehicle- (∼185% greater; *P*<0.001) and cortisol-treated filaments (∼40% lower; *P*=0.006). Similarly, the 53% reduction in *crfbp2* following cortisol treatment (*P*<0.001) was only partially blocked by RU486 co-treatment, as levels of *crfbp2* remained ∼35% lower in cortisol+RU486-treated versus vehicle-treated filaments (*P*=0.006). Finally, cortisol treatment reduced transcript levels of *crfa1* by ∼35% compared with vehicle-treated filaments (*P*=0.03), but this effect was blocked in cortisol+RU486-treated filaments (*P*=0.91).

### Transcriptional effects of CRF peptides in cultured gill filaments

**Fig. 6.**
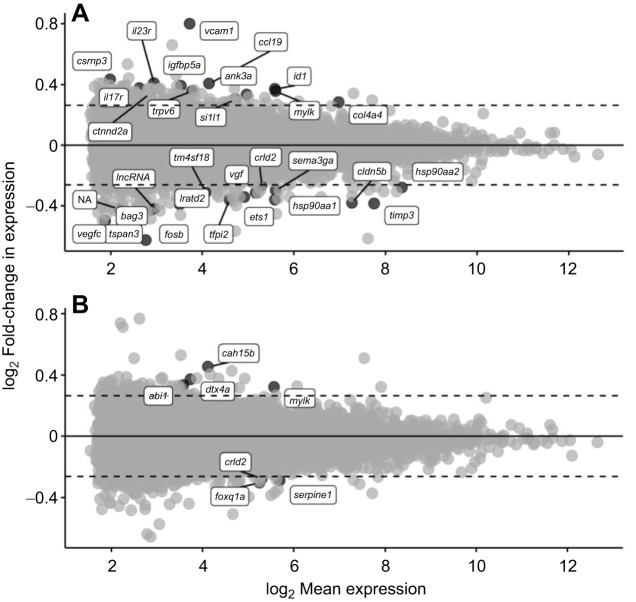
**Transcriptional changes following treatment with CRF peptides.** Effects of CRF a2 (CRFa; A) or urocortin 3 (UCN3; B) treatment on the gill filament transcriptome of FW-acclimated Atlantic salmon (*S. salar*; *N*=8) as determined via RNA-Seq. Treatments were 24 h in duration. Gene names of transcripts (see Table 1 for more details) that showed a ≥20% difference in transcription from vehicle-treated filaments (indicated with dashed lines) and a significant difference following Benjamini–Hochberg correction (*P*<0.05) are shown. Note that data are plotted on a log_2_ scale for visualization purposes.

*In vitro* culture of gill filaments from FW-acclimated salmon in the presence of CRFa2 resulted in 30 differentially expressed genes (DEGs) compared with vehicle-treated filaments; 13 genes were upregulated and 17 genes were downregulated (Fig. 6A, Table 1). Filaments that were cultured with UCN3 – a CRFR2-specific ligand (Manuel et al., 2014) – displayed just seven DEGs compared with vehicle-treated filaments, with four upregulated and three downregulated genes (Fig. 6B, Table 1). Of these DEGs, an upregulation of myosin light chain kinase (*mylk*) was the only change that was statistically significant in both CRFa2- and UCN3-treated filaments following multiple comparison adjustments. In contrast, no DEGs were identified in CRFa2- or UCN3-treated filaments that were collected from SW-acclimated fish. Plasma osmolality values were 5.9% higher in SW-acclimated fish than in FW-acclimated fish, but plasma cortisol levels were not different between FW- and SW-acclimated fish (Table 2).

### Confirmation of RNA-Seq results using qPCR

In general, similar patterns were observed across all genes that were evaluated in both the RNA-Seq analysis and the qPCR follow-up analysis (Fig. S3). However, the only significant differences that were detected via qPCR occurred following CRFa2 treatment. Specifically, transcript levels of *vcam1* (+75%; *P*=0.02), *igfbp5a* (+33%; *P*<0.001), *trpv6* (+31%; *P*=0.004) and *mylk* (+33%; *P*=0.003) were significantly upregulated, while transcript levels of *serpine1* (−25%; *P*=0.02), *hsp90aa1* (−23%; *P*=0.01) and *timp3* (−23%; *P*=0.02) were significantly downregulated.

**Fig. 7.**
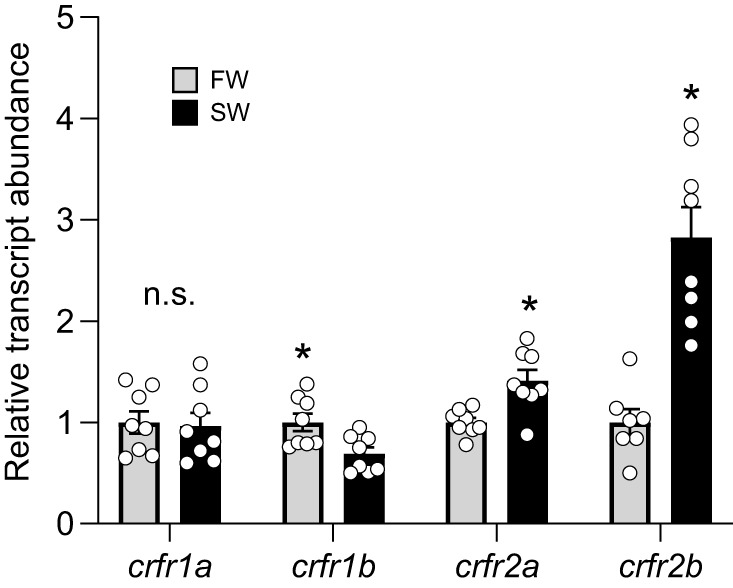
**Changes in corticotropin-releasing factor receptor (CRFR) transcript abundance during tissue culture.** Differences in levels of CRFR 1a (*crfr1a*), 1b (*crfr1b*), 2a (*crfr2a*) and 2b (*crfr2b*) in vehicle-treated gill filaments from FW- (grey) and SW-acclimated (black) Atlantic salmon (*S. salar*). Treatments were 24 h in duration. Significant differences within a gene (*P*<0.05; *t*-test) are depicted using asterisks and data are expressed relative to filaments from FW-acclimated fish. Values are means±s.e.m. and individual data points are shown (*N*=7–8). n.s., no significant difference.

Additionally, transcript abundance of CRFRs in cultured filaments following 24 h in hypoosmotic culture media varied between FW- and SW-acclimated fish (Fig. 7). Levels of *crfr2a* (40%; *P*=0.003) and *crfr2b* (180%; *P*<0.001) were both higher in vehicle-treated filaments from SW-acclimated salmon than in filaments from FW-acclimated salmon. In contrast, transcript levels of *crfr1b* (−30%; *P*=0.01) were lower in vehicle-treated filaments from SW-acclimated fish, but levels of *crfr1a* did not differ between groups (*P*=0.84).

### Determination of CRFR1-specific transcriptional changes

**Fig. 8.**
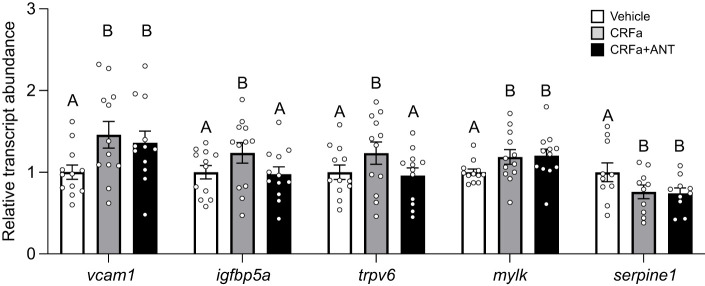
**Determination of receptor-specific effects associated with CRFa.** Changes in levels of vascular cell adhesion molecule 1 (*vcam1*), insulin-like growth factor binding protein 5a (*igfbp5a*), transient receptor potential cation channel V6 (*trpv6*), myosin light chain kinase (*mylk*) and serpin peptidase inhibitor E1 (*serpine1*) following CRFa treatment of gill filaments from FW-acclimated Atlantic salmon (*S. salar*). Treatments were 24 h in duration. Filaments that were treated with vehicle alone, CRFa2 (CRFa) and CRFa2 plus the CRF receptor 1-specific antagonist antalarmin (CRFa+ANT) are depicted in white, grey and black, respectively. Significant differences within a gene (*P*<0.05; one-way ANOVA) are depicted using letters and data are expressed relative to vehicle-treated filaments. Values are means±s.e.m. and individual data points are shown (*N*=10–12).

Some but not all effects of CRFa2 were blocked by antalarmin (Fig. 8). Specifically, the transcriptional effects of CRFa2 on *ifgbp5a* (*P*=0.008) and *trpv6* (*P*=0.003) were both blocked by antalarmin, but CRFa2's effects on *vcam1* (*P*=0.003), *mylk* (*P*=0.003) and *serpine1* (*P*=0.002) were not. Levels of both *igfbp5a* and *trpv6* were 25% higher in CRFa-treated filaments compared with vehicle- (*ifgbp5a*: *P*=0.047; *trpv6*: *P*=0.03) or CRFa+antalarmin-treated filaments (*ifgbp5a*: *P*=0.03; *trpv6*: *P*=0.01), but no differences were detected between vehicle- and CRFa+antalarmin-treated filaments (*ifgbp5a*: *P*=0.96; *trpv6*: *P*=0.88). In contrast, changes in transcript abundance of *vcam1* (+46%), *mylk* (+24%) and *serpine1* (−24%) compared with vehicle-treated filaments were detected when comparing against either CRFa- (*vcam1*: *P*=0.01; *mylk*: *P*=0.03; *serpine1*: *P*=0.03) or CRFa+antalarmin-treated filaments (*vcam1*: *P*=0.046; *mylk*: *P*=0.02; *serpine1*: *P*=0.02), while no differences were detected between CRFa- and CRFa+antalarmin-treated filaments (*vcam1*: *P*=0.77; *mylk*: *P*=0.94; *serpine1*: *P*=0.97). No treatment effects were detected for *timp3* (*P*=0.66) or *hsp90aa1* (*P*=0.58) in this landlocked source population (data not shown).

## DISCUSSION

The CRF system is mainly recognized as the primary neural regulator of cortisol synthesis (Best et al., 2024), but the CRF system also has important physiological functions outside the brain. Here, we show that most components of the CRF system (including ligands, receptors and binding proteins) are expressed in the gills of Atlantic salmon, and that transcript abundance of several components changes during the parr-to-smolt transformation, as well as following changes in environmental salinity (e.g. transfer from FW to SW or SW to FW). Specifically, transcript levels of CRF ligands (*crfa1*, *crfa2* and *crfb2*) were elevated in smolts compared with parr, whereas levels of *crfb2*, *ucn2a*, *crfr2a* and *crfbp2* decreased following SW transfer. Similarly, transfer from SW to FW stimulated the transcription of *crfr2a* and *ucn2a*, and CRFR2 abundance (both *crfr2a* and *crfr2b*) increased when gill filaments from SW-acclimated fish were held in hypoosmotic culture media for 24 h. These changes did not appear to be mediated by interactions with cortisol as cortisol-treated gill filaments exhibited changes that were different from those observed following either FW–SW or SW–FW transfer. Instead, these changes reflect direct contributions by the CRF system as indicated by RNA-Seq analysis of CRF-treated gill filaments. Overall, our results support a direct osmoregulatory role for the CRF system in the gills of Atlantic salmon.

As in mammals and insects, the CRF system appears to promote ion and water excretion across osmoregulatory tissues in fish (Chan, 1975; Loretz et al., 1981; Mainoya and Bern, 1982; Marshall and Bern, 1981). Acquisition of SW tolerance during smoltification is largely mediated by the transition from ion absorption to ion excretion across the gills (Nance et al., 1990; Primmett et al., 1988), whereas water permeability is only marginally lower in SW- versus FW-acclimated fish (Evans, 1984). Thus, transcriptional activation of the gill CRF system in smolts (as indicated by elevated levels *crfa1*, *crfa2* and *crfb2*) could be related to the development of ion secretory mechanisms in the gills but is less likely to be related to changes in water transport. Indeed, activation of the CRF system has previously been shown to facilitate Na^+^ and Cl^−^ transport across various epithelial tissues in fish (Loretz et al., 1981; Mainoya and Bern, 1982; Marshall and Bern, 1981). Cortisol and several other hormones can exert similar stimulatory effects on Na^+^ and Cl^−^ secretion across the gills (Takei et al., 2014) and it is possible that the CRF system interacts with these hormone systems to affect osmoregulatory processes *in vivo*. However, if activation of the gill CRF system is directly involved in the stimulation of ion secretion across the gills, then it is unclear why CRF system activity would decrease (lower levels of *crfb2*, *crfbp2*, *crfr2a* and *ucn2a*) following transfer from FW to SW and increase (higher levels of *crfr2a* and *ucn2a*) following transfer from SW to FW, although this may reflect different roles for the two CRFR subtypes in the gills. In support of this idea, chronic acclimation (30 days) of the FW euryhaline Mozambique tilapia (*Oreochromis mossambicus*) to SW was associated with a >5-fold increase in gill transcript abundance of both *crfb* and *crfr1* (Aruna et al., 2012). This suggests that gill CRFR1 activity may be stimulated in SW, in contrast to the downregulation of gill CRFR2 activity that we observed during SW acclimation of anadromous Atlantic salmon. We also observed elevated levels of CRFR1 (*crfr1b*) in the gills 10 days after juvenile Atlantic salmon were transferred to SW – and levels of *crfr1b* were lower in gill filaments originating from SW- versus FW-acclimated fish following 24 h in hypoosmotic culture media – but we did not detect any changes in CRFR1 levels when SW-acclimated fish were transferred to FW. Additionally, levels of CRFb (*crfb2*) were generally lower in SW- compared with FW-acclimated fish across all our experiments indicating that different regulatory mechanisms may exist between species. Indeed, changes in the gill CRF system of the marine euryhaline black porgy following transfer to FW [i.e. transient elevation of *crfr1* at 24 h post-transfer coupled with a transient reduction of *crfb* at 24 and 96 h post-transfer (Aruna et al., 2021)] were distinct from what we observed following SW-to-FW transfer of Atlantic salmon. Thus, while the CRF system is clearly implicated in responses to osmotic disturbances, specific responses of the gill CRF system following changes in the osmotic environment potentially vary across species.

Despite cortisol being a major regulator of the central CRF system (Aguilera, 1998; Bernier et al., 2009; Best et al., 2024) – and our finding that cortisol transcriptionally regulates many components of the gill CRF system – it is unlikely that the changes observed during our *in vivo* experiments simply reflect changes in cortisol signalling. While transfer of juvenile Atlantic salmon from FW to SW stimulated cortisol production and reduced levels of *crfr2a*, *ucn2a* and *crfbp2*, treatment of cultured gill filaments with cortisol was only able to replicate the reduction of *crfbp2*. In fact, cortisol treatment stimulated transcript abundance of CRFR2 (*crfr2a* and *crfr2b*) via glucocorticoid receptor-mediated signalling pathways, counter to the reduction observed during our *in vivo* experiments. In contrast, all other cortisol-mediated effects on transcript abundance of gill CRF system components were suppressive (with mixed involvement of glucocorticoid receptor-signalling pathways). Interestingly, Aruna et al. (2021) found that gill filaments of marine black porgy had higher levels of *crfb* and *crfr1* when cultured filaments were treated with the synthetic corticosteroid dexamethasone. In mammals, the suppressive action of corticosteroids on CRF transcription in the paraventricular nucleus of the hypothalamus is mediated by a negative glucocorticoid response element (nGRE) in the CRF promoter (King et al., 2002; Makino et al., 1994; Malkoski and Dorin, 1999). However, corticosteroids stimulate CRF transcription in other regions of the brain (e.g. amygdala and bed nucleus of the stria terminalis) and the placenta via interactions with a cAMP response element (CRE) in the CRF promoter (King et al., 2002; Makino et al., 1994; Robinson et al., 1988). Therefore, it is possible that the divergent responses observed in Atlantic salmon and black porgy reflect species-specific differences in the presence of CREs/nGREs in the promoter of different CRF system components and/or differences in the relative abundance of certain transcription factors (e.g. CRE binding proteins). Additional research will help to determine which regulatory mechanism is more prominent in the gills across teleosts.

While the gill CRF system has previously been shown to be affected by the osmotic environment (Aruna et al., 2012, 2021), specific physiological functions of the CRF system in the gills have not been determined. Here, we found that treatment of cultured gill filaments with CRFa2 had transcriptional effects on several genes involved in the regulation of endothelial permeability (*ctnnd2a*, *cldn5b*, *inavaa*, *mylk*, *sema3ga* and *vcam1*). The endothelium regulates vascular integrity, and ultimately controls the exchange of solutes and water across the endothelial barrier (Cerutti and Ridley, 2017). An endothelial CRF system is present in mammals (Fleisher-Berkovich et al., 1998) and while we do not yet know where different components of the CRF system are cellularly located in the gills of Atlantic salmon, CRF-immunoreactive cells are present in the gill filament endothelium of common carp (Mazon et al., 2006). In mammals, activation of CRFR1 in the skin promotes vasodilation and vascular permeability via a mast cell-dependent mechanism (Theoharides et al., 1998). In contrast, increases in the permeability of endothelial cells from human umbilical veins are regulated by CRFR2-mediated disruption of the cadherin–catenin complex, which promotes the dissociation of VE-cadherin and β-catenin (Wan et al., 2013). We found that CRF-mediated increases in transcript levels of *vcam* and *mylk* – both of which help to regulate endothelial permeability (Cerutti and Ridley, 2017) – were not blocked by the CRFR1-specific antagonist antalarmin. Additionally, levels of *mylk* were upregulated following treatment with either CRFa2 and UCN3, suggesting that CRFR2 plays a greater role in regulating endothelial permeability in fish gills. As *crfr2a* and *ucn2a* (a CRFR2-specific ligand) were the two CRF system components which were most affected by changes in the osmotic environment, it is possible that these transcriptional effects are linked to differential regulation of endothelial permeability in the gill filaments of FW- versus SW-acclimated salmon (e.g. movement of solutes).

Modifications to transport rates of solutes and/or water across the gills could similarly be regulated by changing blood vessel density via angiogenesis, whereby greater blood flow could allow for a higher exchange capacity. In mammals, the two CRFR subtypes have opposing effects on angiogenesis wherein CRFR1 promotes angiogenesis and CRFR2 supresses it (Bale et al., 2002; Im et al., 2010). While the current data suggest that the CRF system transcriptionally influences genes related to angiogenesis in the gills of Atlantic salmon (*vegfc*, *ets1*, *ilr17e*, *sema3ga* and *tm4sf18*), we are not aware of any other study that has evaluated a role of the CRF on angiogenesis in fish. Thus, further research is needed to better understand the mechanisms underlying interactions between CRF and angiogenesis in fish. However, the relationships between CRF and endothelial permeability/angiogenesis observed in the current study, combined with previous work showing that UTS1 has vasodilatory effects in rainbow trout (Mimassi et al., 2000), clearly support the vasculature as a major target of the CRF system in fishes, as in mammals.

Changes in endothelial permeability are often associated with parallel changes in inflammation (Cerutti and Ridley, 2017), and we also detected several effects of CRF treatment on immune-related genes in the gills. Specifically, we observed elevated levels of interleukin 23 receptor alpha (*ilr23a*), interleukin 17 receptor E (*ilr17e*) and chemokine (C-C motif) ligand 19a (*ccl19a*) in CRFa2-treated filaments from FW-acclimated fish, indicating that CRF system activation stimulated immune activity in the gill. Notably, activation of IL23 and IL17 pathways is linked with diseases related to inflammation and barrier dysfunction in the intestine of mammals [e.g. irritable bowel disease (Maloy, 2008)], and these same diseases are influenced by CRFR1 activity (Chang et al., 2011; Stengel and Taché, 2009). In salmon, immune function is broadly reduced following SW transfer (Johansson et al., 2016), and it is therefore possible that the general downregulation of CRF system components observed following SW transfer is, at least in part, related to reductions in immune responsiveness. Indeed, the lack of response to CRF treatment in gill filaments from SW-acclimated fish – which was run in parallel to FW filaments during both the experiment itself and subsequent RNA-Seq analysis – is consistent with this hypothesis. In general, the mammalian CRF system has proinflammatory effects that are mediated in part by both CRFR1 and CRFR2 (Baigent, 2001; Karalis et al., 1997), with several of these effects driven by interactions with mast cells (Castagliuolo et al., 1996; Elenkov et al., 1999). We are aware of only a few studies which have investigated direct interactions between immune challenges and the CRF system in peripheral tissues of fish. In ayu (*Plecoglossus altivelis*), UTS1 treatment improved survivorship following a bacterial challenge and *in vitro* treatment of head kidney-derived monocytes/macrophages with UTS1 enhanced rates of phagocytosis and bacterial clearance (Jiang et al., 2021). In contrast, Singh and Rai (2011) found that treatment of spleen phagocytes with UTS1 reduced rates of phagocytosis, but increased superoxide production via a CRFR1-mediated mechanism in spotted snakehead (*Channa punctata*). Lastly, Mazon et al. (2006) reported that CRF and CRFBP were present in macrophage-like cells in the skin and gills of common carp (*Cyprinus carpio*) and chronic parasite infection reduced levels of *crfr1* and *crfbp* in the gills, but not the skin. While we did not directly evaluate which CRFR mediated the effect of CRFa2 on immune-related genes (their levels were relatively low in these non-immune challenged fish), the results of these previous studies combined with the lack of change in any immune-related genes following treatment with UCN3 indicates that CRFR1 may be more important than CRFR2 for coordinating immune responses in fish. However, while it is apparent that the CRF system has conserved immunoregulatory functions in mammals and fish, additional studies are needed to determine the extent to which the mechanisms underlying these interactions are conserved in teleosts.

In conclusion, we have conducted the most thorough investigation into the physiological roles of the CRF system in the gills of any fish species to date. We found that transcript levels of the gill CRF system change in advance of seasonal migrations and respond following perturbations in the osmotic environment in anadromous Atlantic salmon. Specifically, the gill CRF system generally appears to be more active under hypoosmotic conditions; however, confirmation that these transcriptional responses reflect changes at the protein level are needed. These changes in gill CRF system activity appear to contribute to the regulation of endothelial permeability, angiogenesis and immune function in the gills. Overall, our data shed additional light on the peripheral functions of the CRF system and further our understanding of how fish cope with osmotic disturbances.

## Supplementary Material

10.1242/jexbio.248168_sup1 Supplementary information
