# Supplementary material for: Regulation and function of the gill corticotropin-releasing factor system during osmoregulatory disturbances in Atlantic salmon
Source: J Exp Biol. 2025 Jan 28;228(2):JEB248168. doi: 10.1242/jeb.248168 (PMC11832130; doi:10.1242/jeb.248168)
Supplement: Supplementary information [file jexbio-228-248168-s1.pdf]

Supplementary Materials and Methods

Measurement of CRF-induced PKA substrate phosphorylation as determined by SDS-PAGE and western blotting

To determine the dose of CRF peptides to use in our *in vitro* setup, we initially evaluated how different levels of CRFa2 affected phosphorylation levels of PKA substrates. Like CRFb, CRFa is a potent stimulator of CRFR1 in teleosts (Hosono et al., 2015; Manuel et al., 2014; Pohl et al., 2001) and one of the major effectors of CRFR1's actions is protein kinase A (PKA) (Aguilera et al., 1983; Papadopoulou et al., 2004; Pohl et al., 2001). Therefore, we used PKA-substrate phosphorylation as an indication that CRFa2 was exerting physiological effects in cultured gill filaments from freshwater acclimated fish (N = 6; Length: 31.0 ± 0.4 cm; Mass: 347.3 ± 20.6 g; Average ± SEM).

Filaments were incubated in 0.5 mL of MEM containing either 0.1% DMSO (vehicle control) or 10^{-6}, 10^{-8}, or 10^{-10} M CRFa2. We also incubated filaments in 0.5 mL of MEM that contained either 0.1% ethanol (vehicle control) or 12 µM Forskolin (Product #11018; Cayman Chemical Company), as a positive control (N = 4; Length: 30.8 ± 1.2 cm; Mass: 289.3 ± 22.9 g). Filaments were left in these solutions for either 1- or 24-hours, after which the filaments were collected and immediately frozen on dry ice. The filaments were then homogenized using a Precellys Evolution bead homogenizer (Bertin Instruments) in radioimmunoprecipitation assay (RIPA) buffer (150 mM NaCl, 1% triton X-100, 0.5% sodium deoxycholate, 0.1% SDS, 50 mM Tris-HCl) that contained protease (0.574 mM phenylmethylsulfonyl fluoride, 2 mM ethylenediaminetetraacetic acid) and phosphatase (100 mM sodium fluoride, 4 mM tetrasodium pyrophosphate, 2 mM sodium orthovanadate) inhibitors. Protein concentrations were determined

Journal of Experimental Biology • Supplementary information

using Bradford assays (Bio-Rad Protein Assay Dye Reagent, Bio-Rad) with bovine serum albumin as standard.

Homogenates (30 µg) were diluted in 4x Laemmli buffer, heated at 75°C for 10 min and run on a 10% SDS-polyacrylamide gel (with 4% stacking gel) with a protein ladder (PageRuler prestained protein ladder; Thermo Scientific). We also included a calibrator sample of pooled gill protein to normalize values across gels. Gels were run at 100 V for 30 min, followed by ~1 h at 150V (or until the dye front reached the edge of the gel). Proteins were then transferred from the gel to a polyvinylidene difluoride (PVDF) membrane (0.45µm; Immobilon-P) with transfer buffer (25 mM Tris, 192 mM glycine, 15% methanol) using a wet transfer unit (Min-Protean Tetra, Bio-Rad) at 100 V for 1 h. The blot was blocked with 5% BSA in Tris-buffered saline with Tween (TBST; 150 mM NaCl, 20 mM Tris, 0.05% Tween) for 1 h at room temperature (RT). All blocking and incubation steps were performed with shaking. Blots were incubated at 4°C overnight with primary antibody (1:10,000 Phospho-PKA Substrate Rabbit mAb; Product #9624S, Lot #21, Cell Signaling Technology) in TBST with 5% BSA and 0.05% sodium azide. This antibody has previously been validated to measure PKA activity in salmonids (e.g., Best and Gilmour, 2022; Dindia et al., 2013; Forbes et al., 2019). The following morning, blots were washed 3 × 5 mins in TBST, and then incubated with polyclonal goat anti-rabbit horseradish peroxidase (HRP) secondary antibody (1:20,000 in TBST with 5% BSA; Product #AS09602, Agrisera) for 1 h at RT. Blots were washed 3 × 5 min in TBST, 1 x 5 min in TBS, and then chemiluminescent detection of protein bands was performed using Superbright ECL (Product #AS16ECL-S, Agrisera). Blots were imaged using a Bio-Rad ChemiDoc MP Imaging System (Universal Hood III, Bio-Rad) and analyzed by measuring background adjusted lane volumes using ImageLab (Bio-Rad; Version 6.0.1), as described by (Taylor and Posch, 2014). Total PKA-

Journal of Experimental Biology • Supplementary information

substrate phosphorylation was normalized to background adjusted total protein lane volumes, which were determined using Coomassie staining (see Welinder and Ekblad, 2011).

Phosphorylation of PKA substrates by CRF

Treatment of cultured gill filaments with forskolin caused marked increases in the phosphorylation status of PKA substrates compared to vehicle-treated filaments after either 1h (195%) or 24h (185%; Supp. Fig 1). The highest levels of phosphorylation observed in filaments that were cultured with CRFa occurred at 10^{-10} M, with levels being 21% (1h) and 82% (24h) greater than vehicle-treated filaments. Increasing concentrations of CRFa resulted in a dose-dependent reduction of PKA substrate phosphorylation compared to vehicle-treated controls at both 10^{-8} M (15% and 55%) and 10^{-6} M (-19% and 20%). Based on these results, as well as previous studies investigating activation thresholds for CRF-Rs in fish (Hosono et al., 2015; Manuel et al., 2014; Pohl et al., 2001), we treated filaments with 10^{-9} M (~5 ng mL^{-1}) for our RNA-Seq experiments.

Journal of Experimental Biology • Supplementary information

References

Aguilera, G., Harwood, J. P., Wilson, J. X., Morell, J., Brown, J. H. and Catt, K. J. (1983). Mechanisms of action of corticotropin-releasing factor and other regulators of corticotropin release in rat pituitary cells. *J. Biol. Chem.* **258**, 8039–8045.

Best, C. and Gilmour, K. M. (2022). Regulation of cortisol production during chronic social stress in rainbow trout. *Gen. Comp. Endocrinol.* **325**, 114056.

Culbert, B. M., Regish, A. M., Hall, D. J., McCormick, S. D. and Bernier, N. J. (2022). Neuroendocrine regulation of plasma cortisol levels during smoltification and seawater acclimation of Atlantic salmon. *Front. Endocrinol.* **13**, 859817.

Dindia, L., Faught, E., Leonenko, Z., Thomas, R. and Vijayan, M. M. (2013). Rapid cortisol signaling in response to acute stress involves changes in plasma membrane order in rainbow trout liver. *Am. J. Physiol.* **304**, E1157–E1166.

Forbes, J. L. I., Kostyniuk, D. J., Mennigen, J. A. and Weber, J.-M. (2019). Glucagon regulation of carbohydrate metabolism in rainbow trout: in vivo glucose fluxes and gene expression. *J. Exp. Biol.* **222**, 211730.

Hosono, K., Kikuchi, Y., Miyanishi, H., Hiraki-Kajiyama, T., Takeuchi, A., Nakasone, K., Maehiro, S. and Okubo, K. (2015). Teleocortin: A novel member of the CRH family in teleost fish. *Endocrinology* **156**, 2949–2957.

Manuel, R., Metz, J. R., Flik, G., Vale, W. W. and Huising, M. O. (2014). Corticotropin-releasing factor-binding protein (CRF-BP) inhibits CRF- and urotensin-I-mediated activation of CRF receptor-1 and -2 in common carp. *Gen. Comp. Endocrinol.* **202**, 69–75.

Papadopoulou, N., Chen, J., Randeva, H. S., Levine, M. A., Hillhouse, E. W. and Grammatopoulos, D. K. (2004). Protein kinase A-induced negative regulation of the corticotropin- releasing hormone R1α receptor-extracellularly regulated kinase signal transduction pathway: The critical role of Ser301 for signaling switch and selectivity. *Mol. Endocrinol.* **18**, 624–639.

Pohl, S., Darlison, M. G., Clarke, W. C., Lederis, K. and Richter, D. (2001). Cloning and functional pharmacology of two corticotropin-releasing factor receptors from a teleost fish. *Eur. J. Pharmacol.* **430**, 193–202.

Taylor, S. C. and Posch, A. (2014). The design of a quantitative western blot experiment. *Biomed. Res. Int.* **2014**, 361590.

Welinder, C. and Ekblad, L. (2011). Coomassie staining as loading control in Western blot analysis. *J. Proteome Res.* **10**, 1416–1419.

Journal of Experimental Biology • Supplementary information

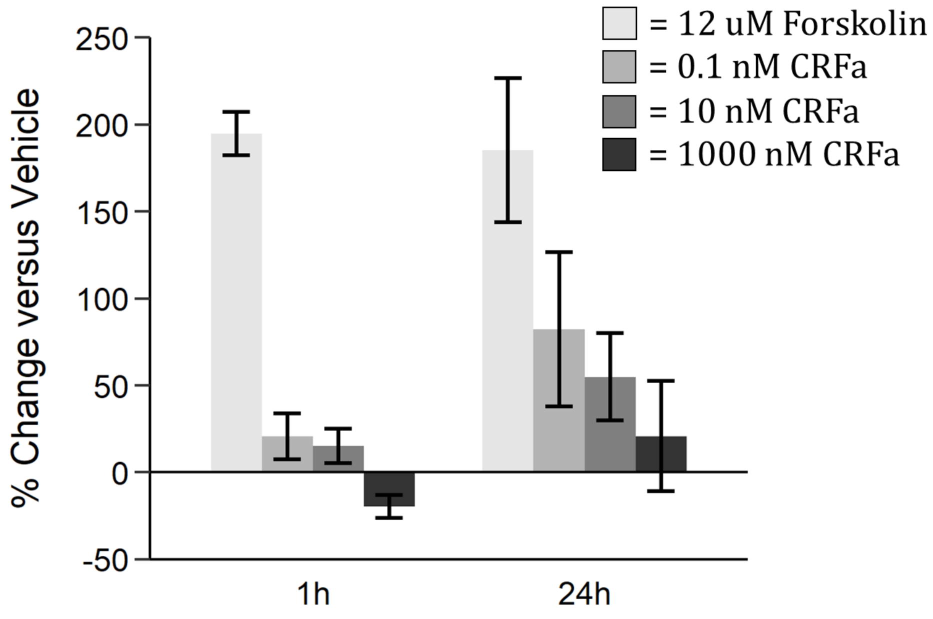

Fig. S1. Effects of corticotropin-releasing factor a2 (CRFa) on gill protein kinase A (PKA) activity. Dose-dependent effects of CRFa on relative protein abundance of phosphorylated PKA substrates in the gills of freshwater-acclimated Atlantic salmon (*Salmo salar*). The adenylate cyclase agonist forskolin was included as a positive control. Data are expressed as percent difference from vehicle-treated filaments and values are represented as means ± SEM (N = 4 for forskolin and N = 6 for CRFa).

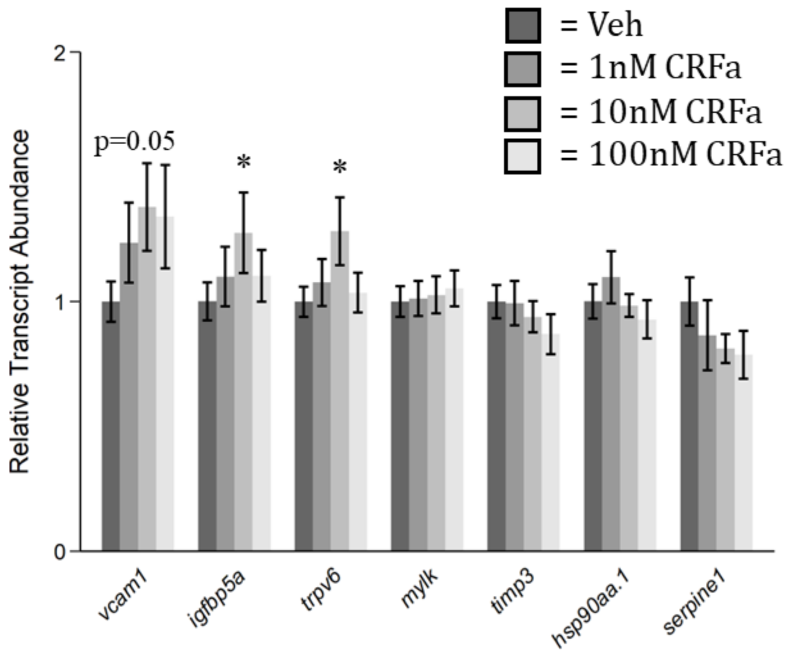

Fig. S2. Effects of corticotropin-releasing factor a2 (CRFa) on transcript abundance of target genes in the gills. Dose-dependent effects of CRFa on levels of vascular cell adhesion molecule 1 (*vcam1*), insulin-like growth factor binding protein 5a (*igfbp5a*), transient receptor potential cation channel V6 (*trpv6*), myosin light chain kinase (*mylk*), metalloproteinase inhibitor 3 (*timp3*), heat shock protein 90aa (*hsp90aa1*), and serine protease inhibitor E1 (*serpine1*) in the gills of freshwater-acclimated Atlantic salmon (*Salmo salar*). Significant differences within a gene ($p < 0.05$) are depicted using asterisks and data are expressed relative to vehicle (Veh)-treated filaments. Values are represented as means ± SEM (N = 12).

Journal of Experimental Biology • Supplementary information

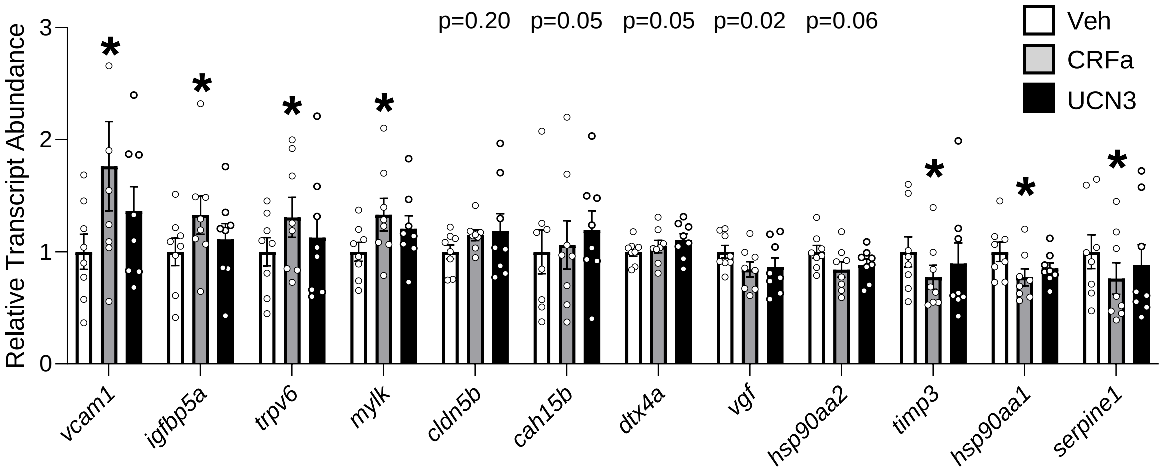

Fig. S3. Confirmation of corticotropin-releasing factor a2 (CRFa) and urocortin 3 (UCN3) effects on target genes in the gills. Effects of CRFa and UCN3 on levels of vascular cell adhesion molecule 1 (*vcam1*), insulin-like growth factor binding protein 5a (*igfbp5a*), transient receptor potential cation channel V6 (*trpv6*), myosin light chain kinase (*mylk*), claudin 5b (*cldn5b*), carbonic anhydrase 15b (*cah15b*), deltex 4 E3 ubiquitin ligase a (*dtx4a*), VGF nerve growth factor inducible (*vgf*), heat shock protein 90aa (*hsp90aa1*/*hsp90aa2*), metalloproteinase inhibitor 3 (*timp3*), and serpin peptidase inhibitor E1 (*serpine1*) in the gills of freshwater-acclimated Atlantic salmon (*Salmo salar*). Treatments were 24 h in duration. Filaments treated with vehicle alone (Veh), CRFa2 (CRFa), and UCN3a/b (UCN3) are depicted in white, grey, and black, respectively. Significant differences ($p < 0.05$; one-way ANOVA) between the vehicle group and one of the treatment groups are depicted using an asterisk. Data are expressed relative to vehicle-treated filaments. Values are represented as means ± SEM and individual data points are shown (N=8). P-values of the overall model are shown in instances where significant differences were not detected according to either the results of the overall model (*cldn5b*, *cah15b*, *dtx4a*, and *hsp90aa2*) or subsequent post hoc analysis (*vgf*).

Journal of Experimental Biology • Supplementary information

Table S1. Gene specific primers used for real-time polymerase chain reaction (qPCR).

	Primer Sequence (5' to 3')	Amplicon Size (bp)	Efficiency (%)	Accession Number	Reference
cah15b	F: GGAGGTGTTGGCTTTAGGGA<br>R: AGTGGGGACAAAGAGTGTCG	103	107	XM_014140878	Current Study
cldn5b	F: AAGGAGCGAAGATGGTGTCC<br>R: ATAACGCACAGTCCCAGTCC	58	109	XM_014170805	Current Study
crfa1	F: TCGCCGAACACATCTCCTG<br>R: TGCGTGAGCTGAAGTTGTAA	74	101	XM_014139988	Culbert et al., 2022
crfa2	F: GGTCAACAGGGCTTTACTTCA<br>R: AACCGATTGCTGTTACCGAC	69	103	XM_014190344	Culbert et al., 2022
crfb1	F: CTTGATCCATCACTCGTGGAAA<br>R: GTCAGGGGTTCAACGAGATC	98	108	XM_014181363	Culbert et al., 2022
crfb2	F: GAGGAAGGCAGCTCTCAACT<br>R: TCATGTCGGGATCAACAGGAA	84	96	XM_014159556	Culbert et al., 2022
crfbp1	F: GTTTCTGAAGGGAGACACCAGA<br>R: TCGCCTGTAAATGAATGTCTCG	118	107	XM_014128333	Current Study
crfbp2	F: GTGAGGGTGTTAGCAGGGTG<br>R: CCACAGTCCCAAAACGACAC	116	103	NM_001173799	Current Study
crfr1a	F: AGCTACAGAGGCTGACAATGG<br>R: CTGTGCTGCCTTTGGCAAAG	69	99	XM_014204418	Culbert et al., 2022
crfr1b	F: CAGAGGGTCAGGATGACAATG<br>R: GTGGCAAAAAGTCAGCTCTTG	59	108	XM_014192620	Culbert et al., 2022
crfr2a	F: ACCTGGATCGCCAAGTTCA<br>R: AGTGCTCTGTCTCTGTCTTCA	141	107	XM_014140566	Current Study
crfr2b	F: GACCAAATCCAAAGGTCACCT<br>R: GCTCTCTGTCCTTATGCAGC	123	99	XM_014191256	Current Study
dtx4a	F: GACACCAGCTTCATTCGTCTC<br>R: TCCGACCTAACACAAACCGA	130	103	XM_014155723	Current Study
ef1α	F: TCCTGCGGAGTCTCAAAACC	96	103	XM_014141923	Culbert et al., 2022

Journal of Experimental Biology • Supplementary information

	R: CGTTGGGTTCTTTTCCTGCG				
hsp90aa1	F: ATGGCAGTTAGCTTTGTCGC	103	105	XM_014205880	Current Study
	R: AGCGGGTCAAGAGATTTTGGA				
hsp90aa2	F: CAGGATGGAGGAGGTTGACT	184	106	NM_001173702	Current Study
	R: ACTGTATGTGACCAATGCGG				
igfbp5a	F: AGGGACTTTTACGCACGCA	87	104	XM_014152143	Current Study
	R: AATGCTTTCGATCATTGGGGT				
mylk	F: CGCTATTTTGGCAACGTGCT	50	106	XM_014164223	Current Study
	R: TGTGTGCGTCCCTCCTTTTT				
nkaα1b	F: GAGGTTGGGTGGAACAGGAG	92	106	XM_014150738	Current Study
	R: TGTTGGTTGAGATGTAGCAGC			XM_014152156	
rpl13a	F: GGACAAGCTGCACTGGAGAG	113	93	XM_014128281	Current Study
	R: GTGGGCTTCAGACGGACAAT				
serpine1	F: CCTCATCCAACACAAGCCCA	143	108	XM_014207192	Current Study
	R: CCTCGCTGGAGAAGACAGAT				
timp3	F: TCCTACGCCCTCTGACGAAA	191	104	XM_014152811	Current Study
	R: AAAGTGGACTGGGATCGGGT				
trpv6	F: GAGAATCGCAACGACCCACT	135	101	XM_045700038	Current Study
	R: GGACTTCCTGTTTCCAGGTCC				
ucn2a	F: CATGCGTCTGTGTCTGAGC	115	93	ENSSSAT00000205481	Current Study
	R: AGCCAACAGGTCACTTTCTG				
ucn2b	F: GCAACCAGTGCCAGATAACG	137	109	XM_014133639	Current Study
	R: GGGTGTTGGATGGAATTCTGG				
ucn3	F: CGCAGATTGGACGAAGGAAG	71	100	XM_045712114	Current Study
	R: ATGGATAATCAGGGTAGCGGC			XM_014185247	
uts1a	F: CAGTGTCTGTAGACCACGG	89	104	XM_014205273	Culbert et al., 2022
	R: TATCACCAGCCTTCAGCAAC				
uts1b	F: GCAGTCTACTACAATCGCCAT	103	101	XM_014144548	Culbert et al., 2022
	R: AAACGGTGCCTTGATCGC				
vcam1	F: GTTTGAGAAGGGGCAGAACG	195	104	XM_014189350	Current Study
	R: AGGCTCTGGTGTCCCACTAC				

vgf	F: GTAAGGAAACCTGCGGTGCT<br>R: GCTCGGTGTTGTTAGGAAGTG	131	109	XM_014207191	Current Study

cah, carbonic anhydrase; *cldn*, claudin; *dtx*, deltex e3 ubiquitin ligase; *crf*, corticotropin-releasing factor; *crfbp*, corticotropin-releasing factor binding protein; *crfr*, corticotropin-releasing factor receptor; *ef1α*, elongation factor 1α; *hsp90*, heat shock protein 90; *igfbp*, insulin-like growth factor binding protein; *mylk*, myosin light chain kinase; *nkaα1b*, sodium potassium ATPase subunit alpha 1b; *rpl13a*, ribosomal protein L13a; *serpine*, serpin peptidase inhibitor clade E; *timp*, tissue inhibitor of metalloproteinase; *trpv*, transient receptor potential cation channel subfamily V; *ucn*, urocortin; *uts*, urotensin; *vcam*, vascular cell adhesion molecule; *vgf*, VGF nerve growth factor inducible.

Journal of Experimental Biology • Supplementary information

Table S2. Number of paired-reads per sample for RNA-Seq analysis on gill filaments collected from freshwater (FW) or seawater (SW) acclimated Atlantic salmon (*Salmo salar*) that were cultured for 24 h in media containing vehicle (Veh), corticotropin-releasing factor A2 (CRFa), or urocortin 3 (UCN3).

	Fish ID	Number of Reads		
FW		Veh	CRFa	UCN3
	1	41,362,092	45,870,794	39,351,061
	2	47,537,502	43,253,980	40,741,961
	3	29,634,786	50,223,930	35,292,821
	4	30,856,135	33,412,580	39,699,471
	5	53,192,825	37,791,770	47,549,763
	6	42,326,219	34,942,405	37,971,303
	7	40,427,744	47,132,685	39,825,472
	8	48,313,644	47,115,687	48,885,292
	Mean	41,706,368	42,467,978	41,164,643
SW		Veh	CRFa	UCN3
	1	40,804,705	35,220,670	34,354,364
	2	39,686,381	43,162,161	46,011,910
	3	39,359,035	37,192,101	46,156,085
	4	43,888,041	31,807,328	43,842,995
	5	40,521,058	37,753,586	39,941,370
	6	49,750,906	34,170,429	45,715,492
	7	49,770,770	40,463,504	38,054,705
	8	60,672,978	43,141,641	44,987,581
	Mean	45,556,734	37,863,927	42,383,062

Journal of Experimental Biology • Supplementary information
